# Harnessing rat derived model cells to assess the toxicity of TiO_2_ nanoparticles

**DOI:** 10.1007/s10856-022-06662-7

**Published:** 2022-05-04

**Authors:** Manizheh Sarikhani, Sevil Vaghefi Moghaddam, Masoumeh Firouzamandi, Marzie Hejazy, Bahareh Rahimi, Hassan Moeini, Effat Alizadeh

**Affiliations:** 1 Department of Medical Biotechnology, Faculty of Advanced Medical Sciences, Tabriz University of Medical Sciences, Tabriz, Iran; 2 Biotechnology Section, Department of Pathobiology, Faculty of Veterinary Medicine, University of Tabriz, Tabriz, Iran; 3 Toxicology Section, Department of Basic Science, Faculty of Veterinary Medicine, University of Tabriz, Tabriz, Iran; 4 Department of Medical Biotechnology, Faculty of Allied Medical Sciences, Iran University of Medical Sciences, Tehran, Iran; 5 Institute of Virology, Faculty of Medicine, Technische Universität of München, Munich, Germany

## Abstract

Until now, a few studies have been conducted on the destructive effects of TiO_2_ NPs in living organisms, and studies on the toxicity of TiO_2_ NPs are still in the beginning phases. Because of the widespread use of TiO_2_ NPs in all areas of human life, it is essential to study their profound and fundamental toxic effects on each organ and body cell. Herein, we evaluate the effect of exposure to TiO_2_ NPs on in vitro models derived from the rat bone marrow and adipose tissues. Exposure to TiO_2_ NPs at 100 and 200 μg/ml exhibited cytotoxicity for the rat bone marrow mesenchymal stem cells (rBMSCs) and rat adipose mesenchymal stem cells (rATSC), respectively. Additionally, reduced rBMSCs and rATSCs frequencies in the S phase of the cell cycle. Moreover, TiO_2_ NPs enhanced the activity of cellular senescence-associated β-galactosidase in both model cells. Significantly higher relative expression of aging-related genes P53 and NF-kB (*p* < 0.05) and lower expression levels of anti-aging-related genes Nanog and SIRT1 were found in the treated cells (*p* < 0.05). Colony-forming and DAPI staining showed the reduction of cell growth and DNA damage in both rBMSCs and rATSCs. Our findings along with other similar findings showed that TiO_2_ NPs probably have negative effects on the cell growth, prompt the cells for entry into proliferation stop, DNA damage, and trigger the aging process.

**Figure:**
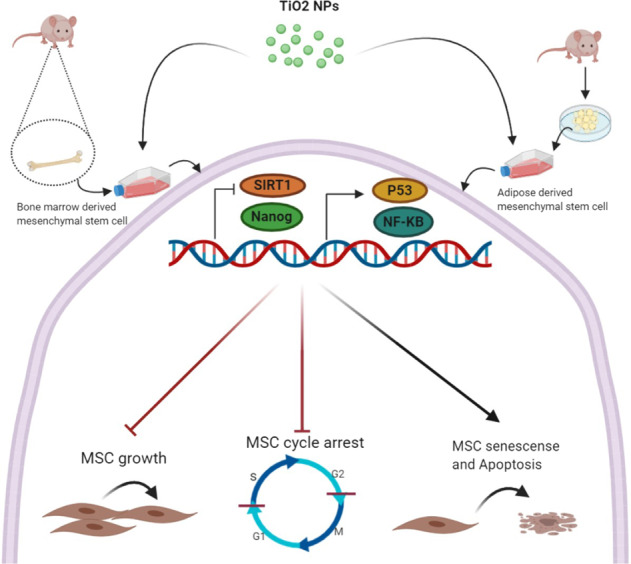
Graphical abstract

Graphical abstract

## Introduction

The advancements in nanotechnology and nanoscience during the last decades revealed innovative and attractive engineered nanoparticles (ENPs) with specific properties in a wide range of areas. In consequence, human beings are intentionally or unintentionally exposed to those ENPs during their lifetime, which leads to increasing concerns in regards to their probable toxicity in human health [1, 2]. On the other hand, due to the unique properties of nano-sized particles they represent more toxicity in comparison to the larger micron-sized particles [3].

Among the existing nanomaterials [4], titanium dioxide nanoparticles (TiO_2_ NPs) are widely used for consumable and industrial applications including coatings, papers, plastics, food products, medicines, pharmaceuticals, toothpaste, and cosmetics [5]. Brookite, anatase, and rutile are three polymorphic forms of TiO_2_ NPs. They indicate a strong refractive index for both UVA and UVB lights and have been universally accepted as a good UV filter [6]. They are prominent for their unique properties such as high stability, large surface area, anti-corrosive, and activities like food colorant and photocatalytic activity. As a result of these predominant properties, the production and application of TiO_2_ NPs are continually increased, leading to progressed human exposure to these nanoparticles.

One of the main problems associated with the application of ENPs such as TiO_2_ NPs is their ability to penetrate through the physiological barriers into the bloodstream and reach the organs such as the kidney, heart, liver, brain, spleen, and bone marrow ([7–10]). In those organs they can pass through the cell membrane and enter the mitochondria, causing organelle dysfunctions [11]. The result of in vitro and in vivo studies demonstrated that the probable toxicity of TiO_2_ NPs could include mitochondrial dysfunction, decretion in cell viability, and generation of oxidative stress causing genotoxicity and apoptosis [12, 13].

The most common way to assess the toxicity of nanoparticles is by investigating their effects on the proliferation of cells. Stem cells are the famous category of proliferative cells. They are positioned in specific niches of a variety of organs and remain quiescent until they receive some signals which activate them to start differentiation or repair [14]. These cells are susceptible to the influence of the particles that enter the cell and even remain in their immediate environment. To prove this fact, we used Mesenchymal stem cells (MSCs) and TiO_2_ NPs in anatase form.

MSCs were used in toxicology research for the first time in1982 [15]. Subsequently, technologies on the stem cell have been directed to various novel developments in toxicity testing [16, 17]. Scanu et al. indicated that human MSCs provided more perfectible in vivo modeling for toxicology research [18]. Therefore, we designed this study to assess the effect of TiO_2_ anatase NPs on stem cells derived from rat bone marrow (rBM) and adipose tissue. Here, we evaluated the effects of TiO_2_NPs on the proliferation, viability, and differentiation potential of human Wharton’s jelly MSCs. Also, the viability, proliferation, cell cycle, apoptosis, senescence gene markers, colony forming, and stemness gene expression in rat bone marrow (rBMSCs) and adipose tissue-originated (rATSCs) cells exposed to TiO_2_ anatase were evaluated. Our work indicated that TiO_2_ NPs could affect both in vitro model cells and prompted cells to reduction of proliferation, colony forming, stemness properties, aging, and apoptosis.

## Material and methods

### Properties of TiO_2_ nanoparticles and preparing the stock solution

Titanium dioxide nanoparticles with 99.5% purity were purchased from Sigma-Aldrich, USA. To prepare 1 mg/ml stock solution, 10 mg of NPs was completely dispersed in the 10 ml routine culture medium. The solution was sterilized by autoclaving and put in the fridge until needed. Then different aliquots (50, 70, 130, 150, 170, 200, 240, and 280 µg/ml) were prepared for the cell culture.

### Field emission scanning electron microscopy (FESEM)

Morphological characterization of the TiO_2_ nanoparticles was studied by field emission scanning electron microscope (Fesem Quantum 200 fei, USA). The samples were prepared by adding TiO_2_ nanoparticles to the propanol (Sigma-Aldrich Co., Ltd.), then suspended using prob type sonicator, and were allowed to dry.

### Dynamic light scattering (DLS)

The particles size distribution of TiO_2_ nanoparticles in the aqueous phase and 25 °C was determined by the DLS technique (Zetasizer Nano ZS90; Malvern Instruments, UK). The samples were prepared at the concentration of 0.1% wt.

### Isolation of MSCs from rat bone marrow or adipose tissue

MSCs were isolated from rBM as previously described by Eslaminejad et al. [19]. Surgery on rats was performed according to ethical considerations by a veterinarian. As a brief protocol, the mice were euthanized in CO_2_ chambers, and two bone types, including tibias and femurs, were dissected. Then, their marrow bones were gently flushed in DMEM (Invitrogen, USA) and supplemented with 10% fetal bovine serum (FBS, Gibco, Germany), 100 U/ml penicillin (Sigma, Germany), and 100 mg/ml streptomycin. The MSCs were isolated by Ficol centrifugation, transferred into a 25-cm^2^ culture vessel, and incubated in an incubator with a standard 37 °C humidified atmosphere containing 5% CO_2_ for 14 days [19]. The extraction of adipose MSCs was conducted as described by Alizadeh et al. [20], with some modifications. Briefly, the subcutaneous adipose tissue was dissected from mice and then minced into tiny fragments. After a while, it was digested with collagenase type I. Then, mononuclear cells, including stromal vascular fraction, were separated using Ficoll (Bioidea, Iran). After that, a mesh filter was used to remove the unwanted cells, and then the RBC lysis buffer was employed to eradicate the residual RBCs. Finally, the extract was transferred to the DMEM culture medium (Gibco, Invitrogen) containing 10% FBS (Gibco, Invitrogen), 1X ITS (Sigma, USA), dexamethasone 1 × 10^−6^ M, and 1% penicillin/streptomycin (Gibco, Invitrogen). After 24 h incubation at 37 °C and 5% CO_2_, the non-adherent cells were removed, and later the medium was refreshed every 3 days until 70–80% confluency was achieved [21]. Osteogenic differentiation and mineralization of the cells were evaluated by alizarin red staining [22].

### Flow cytometry analysis

The CD markers of rBMSCs and rATSCs were further characterized by flow cytometry. After washing with PBS, the cells were stained with FITC-conjugated anti-human CD90 and CD105 (MSC markers), also with CD45 and CD34 (as negative markers) [23]. The cells were then analyzed on a flow cytometer (DB FACS, Dickinson) using FACS CellQuest software for the expression of their surface markers.

### In vitro cytotoxicity assessment

The cytotoxic effect of TiO_2_ NPs on rBMSCs and rATMCs was studied by the MTT (3-(4,5-dimethylthiazol-2-yl)-2, 5-diphenyltetrazolium bromide) (Acros Organics Company) assay. Briefly, the cells were seeded into 96-well plates at a density of 2 × 10^4^ cells/well. After 24 h of incubation at 37 °C, the culture medium was removed, and the cells were treated with different concentrations of TiO_2_ NPs (10–500 µg/ml) in triplicate for 1, 2, and 3 days. Also, untreated cells with 100% viability were used as a control. After completion of the incubation, the culture medium was replaced by a fresh medium containing 20 µl MTT solution (0.5 mg/ml in DMEM). Following 4 h of incubation at 37 °C, intracellular insoluble formazan was solubilized by adding 200 μl/well DMSO (Dimethyl sulfoxide), and absorbance value was measured at 570 nm against a blank reagent using a scanning spectrophotometer (AWARENESS, technology INC). The viability of cells was calculated according to the following formula:

$${{{\mathrm{Cell}}}}\,{{{\mathrm{Viability}}}}\left( \% \right) = \left( {{{{\mathrm{OD}}}}_{{{{\mathrm{treated}}}}} - {{{\mathrm{OD}}}}_{{{{\mathrm{blank}}}}}/{{{\mathrm{OD}}}}_{{{{\mathrm{control}}}}} - {{{\mathrm{OD}}}}_{{{{\mathrm{blank}}}}}} \right) \times 100$$

### Cell cycle assay

rBMSCs and rATSCs were cultured in 6-well plates at an initial cell seeding density of 5 × 10^5^ cells/cm^2^. At confluency of 70%, the cells were treated with TiO_2_ NPs in concentration around their IC_50_ (100 and 200 µg/ml for rBMSCs and rATSCs, respectively). After 72 h, the cells were washed twice with PBS, then fixed with 70% ethanol and stored at 4 °C for 2 days. After collecting the fixed cells by centrifugation, they were washed twice with PBS and resuspended in PBS followed by treated with RNase A for 45 min. Subsequently, DNA intercalating dye propidium iodide solution (PI) (Sigma) was added to the suspension and incubated for 15 min in the dark. The samples were then analyzed by a flow cytometer to determine the percentage of fluorescent intensity. The cell progression in different phases of the cell cycle was evaluated through the DNA histogram by Becton-Dickinson FACS Calibur Flow Cytometer using CellQuest software (Becton-Dickinson, San Jose, CA, USA).

### Apoptosis study by DAPI staining

To visualized the apoptotic effect of TiO_2_ nanoparticles toward rBMSCs and rATSCs, the nuclei of the cells were stained by DAPI (4′,6-diamidino-2-phenylindole) according to the following: the cells were seeded into 6-well plates at the density of 5 × 10^5^ cells/well. After 24 h incubation at 37 °C, the culture medium was changed, and the cells were treated with TiO_2_ NPs in concentration around their IC_50_ (100 and 200 µg/ml for rBMSCs and rATSCs, respectively) for 48 h. Thereafter, the medium was removed, the cells were washed with PBS, and finally fixed with 4% paraformaldehyde for 10 min. The cell permeabilization was carried out by adding a permeabilization buffer (3% paraformaldehyde and 0.5% Triton X-100) and incubating for 15 min. Then the cells’ nuclei were stained with 1 µg/ml DAPI solution (Sigma, Germany). Finally, the cells were resuspended in PBS, and the morphology of the nuclei was assessed under fluorescence microscopy (Olympus IX81, Germany) at ×400 magnification. The condensed and fragmented nuclei were detected in the apoptotic cells.

### Forming-colony assay

We directed colony-forming experiments according to our previously published article [24]. Briefly, rBMSCs or rATSCs in passage 3 were seeded at a density of 200 cells in 100-mm petri dishes in the standard DMEM/FBS medium for 1 day to let the cells adhere. The medium was then replaced by the medium containing TiO_2_ NPs (100 and 200 µg/ml for rBMSCs and rATSCs, respectively) or just the fresh medium in control groups. The colonies were then fixed with 2.5% glutaraldehyde for 20 min. The colonies were stained with 0.5% crystal violet solution for 30 min and finally rinsed with PBS. The colonies were counted using an inverted microscope (Olympus, Center Valley, PA, USA).

### Real-time PCR

Forty-eight hours after treatment with TiO_2_ NPs in concentration around their IC_50_ (100 and 200 µg/ml for rBMSCs and rATSCs, respectively), total RNA was extracted from rBMSCs and rATSCs using an RNA extract kit (Bio Basic INC, USA). cDNA was synthesized using the PrimeScript^TM^ RT Kit (TaKaRa, Japan) and used for real-time PCR using SYBR® Premix Ex Taq™ I (TaKaRa, Japan) and a pair of specific primers for Nanog, P53, SIRT1, NFkb, RUNX2, Osteocalcin, OCT4, and SOX2 genes (Table 1). The real-time PCR reaction was performed in a thermal cycler (BioRad, USA). After initial denaturation at 95 °C for 3 min, amplification was carried out in 40 cycles at 95 °C for 20 s, 60 °C for 20 s, and 72 °C for the 30 s. Relative expression of the genes in each source of the cells was calculated related to the GAPDH housekeeping gene.

**Table 1:** Primer sequences of each gene

Gene	Primer name	Sequence	Length
Nanog	Forward	GAGACTGCCTCTCCTCCGCCTT	22
	Reverse	GTGCACACAACTGGGCCTGA	20
P53	Forward	GTCGGCTCCGACTATACCACTATC	24
	Reverse	CTCTCTTTGCACTCCCTGGGG	21
GAPDH	Forward	TCAAGAAGGTGGTGAAGCAG	20
	Reverse	AGGTGGAAGAATGGGAGTTG	20
NF-KB	Forward	GTCGGCTCCGACTATACCACT	21
	Reverse	TCCTCTCTTTGCACTCCCTGGG	22
Sirt 1	Forward	CGCCTTATCCTCTAGTTCCTGTG	23
	Reverse	CGGTCTGTCAGCATCATCTTCC	22

### In situ assay of β-galactosidase for cellular senescence

Senescence-associated β-galactosidase (SA-β-Gal) staining was conducted utilizing the SA-β-gal staining kit (Dimri, USA) according to the protocol of the manufacturer. Briefly, rBMSCs and rATSCs were cultured in 24-well plates. At the confluency of 80%, the cells were treated with TiO_2_ NPs in concentration around their IC_50_ (100 and 200 µg/ml for rBMSCs and rATSCs, respectively) for 72 h/min. Afterward, the cells were fixed using G/F fixative mix at room temperature for 3–5 min. The cells were washed twice with 1XPBS and stained with the staining solution X-gal at 37 °C for 2 h. The SA-β-gal-positive cells exhibited a green color. Positive cells were counted under an inverted light microscope.

### Statistical analysis

All the experiments were performed in triplicate, and the results were shown as mean ± standard deviation. The experimental data were analyzed by SPSS (IBM Corp. NY, USA) using the two-way analysis of variance.

## Results

### Evaluation of TiO_2_ NPs size by DLS technique

**Fig. 1:**
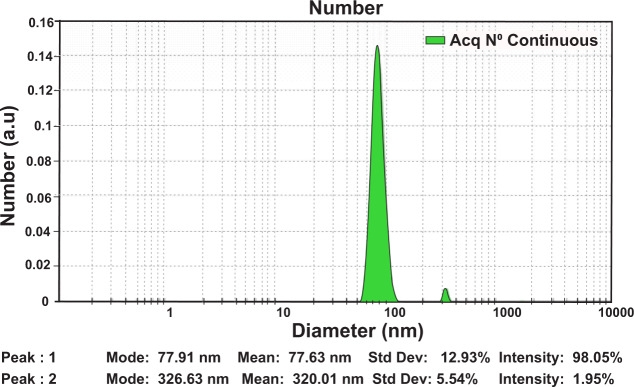
The hydrodynamic size distribution of TiO_2_ (Anatase) NPs at pH 7.4 and temperature 25 °C using the DLS technique

The size distribution of the TiO_2_ NPs was determined by DLS. The results are shown in Fig. 1. To assess the particle size, NPs were redispersed ultrasonically using prob type sonicator (300w, 30 s). As shown in Fig. 1, the majority of NPs (98.05%) was 77.91 nm in size, and an ignorable ratio (about 1.95%) was 326.63 nm. Also, there was a slight increase between the size diameter resulting from the DLS and the primary structure of TiO_2_ NPs resulting from SEM, since in DLS the hydrodynamic diameter of the particles in the liquid is measured.

### SEM images of TiOFE_2_NPs

**Fig. 2:**
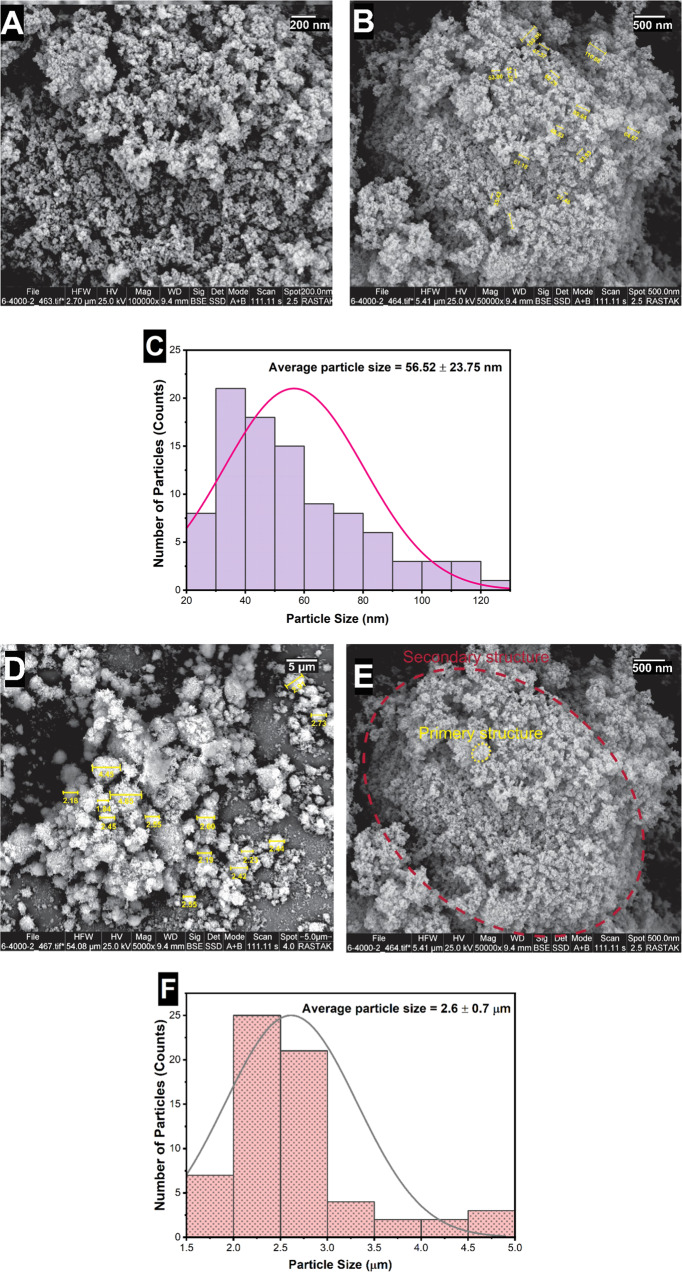
Morphological characterization and size distribution of TiO_2_ NPs. **A**, **B**, **D**, **E** The FESEM micrographs represented the irregular rock-like architectures, which consist of a secondary structure with particle diameters around a few microns (**E** red cycle). And these secondary structures are formed by the agglomeration of the primary structures with particle diameters around a few tens of nanometers (**E** yellow cycle). **C**–**F** The size distribution of the primary and secondary structures of TiO_2_ particles

Figure 2 shows the structure and morphology of the TiO_2_ NPs. As can be seen from the figures, they have an irregular rock-like structure with large variations in size ranging from a few nanometers to around 2.6 microns. The high magnification images of these particles revealed the nanocrystalline domain with an average size of about 56 nm. So, based on the previous works, which confirmed this type of image [25, 26], we can conclude that TiO_2_ NPs consist of a secondary structure with particle diameters around a few microns (Fig. 2E red cycle). And these secondary structures are formed by the agglomeration of the primary structures with particle diameters around a few tens of nanometers (Fig. 2E yellow cycle).

### Characterization of rBMSCs and rATSCs

**Fig. 3:**
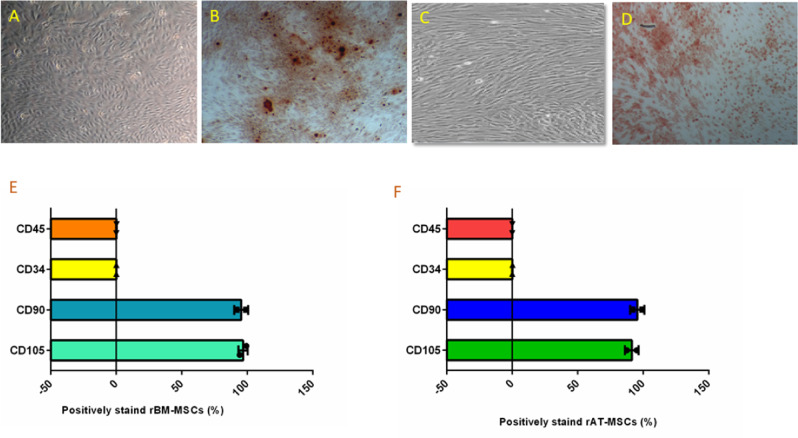
Characterization of rat bone marrow MSCs (rBMSCs) and rat Adipose tissue stem cells (rATSCs). **A** The figure shows rBMSCs, their morphology is spindle shaped, and they established an adherent culture. **B** The figure shows rBMSCs after osteogenic differentiation and subsequently alizarin red staining, presence of red-stained mineralization particles confirmed the osteogenesis potential of them. **C** The figure shows rATSCs, they are a little longer or bigger than rBMSCs when compared under an inverted microscope. **D** The osteogenic induction of rATSCs was directed then the alizarin red staining was performed, presence of red-spots after alizarin red staining showed osteogenesis potential of rATSCs. **E**, **F** The surface markers of rATSCs and rBMSCs were checked using immunophenotyping. The graphs showed that a majority of rBMSCs or rATSCs (95.47% or 95.38%) showed positive surface expression for CD90 as a surface marker in MSCs. Also, 96.77% or 87.56% of them (rBMSCs or rATSCs) were positively stained for CD105. Inversley, a very few percentage (less than 1%) of rBMSCs or rATSCs showed the expression of CD34 and CD45, which was assumed as negative markers

The rat mesenchymal stem cells were isolated from bone marrow and adipose tissue. rBMSCs and rATSCs showed the classic morphology of MSCs, spindle shape with the adherent phenotype (Fig. 3A, C). In initial subcultures, rBMSCs represented smaller cells (Fig. 3A), however, due to the passages giant cells that appeared in the culture, which are the signs of in vitro senescence, rATSCs were a little bigger or longer than rBMSCs, as depicted in Fig. 3C. Alizarin red staining showed the presence of mineralized particles in the stem cells, indicating the osteogenesis potential of the cells (Fig. 3B, D). The majority of rBMSCs or rATSCs (95–97.3%) showed the positive surface expression of CD90 as a surface marker in the MSCs. The rBMSCs and rATSCs were found to be 96.77% or 87.56% positive for CD105, respectively (Fig. 3E, F). Moreover, an ignorable percentage (<1%) of both cells showed the expression of CD34 and CD45 as hematopoietic lineage markers (Fig. 3E, F).

### In vitro cytotoxicity evaluation of TiO_2_ NPs

**Fig. 4:**
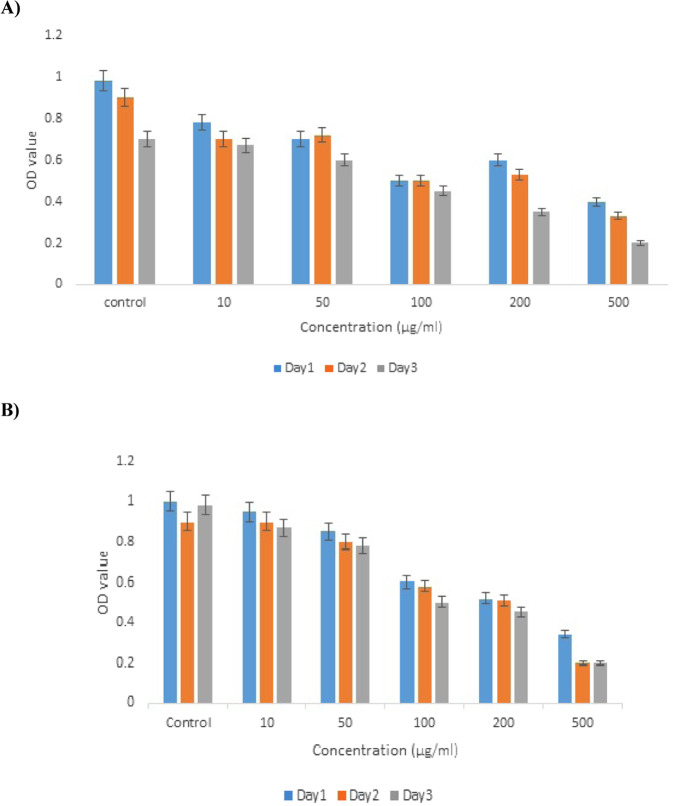
The effects of TiO_2_ NPs on the viability of rBMSCs and rATSCs determined by MTT assay after treating with (0–500 µg/ml) TiO_2_ NPs for 1, 2, and 3 days in comparison to control group. **A** In the rBMSCs, the inhibitory concentration in half maximum (IC_50_) was around 100 µg/ml TiO_2_ NPs. **B** In the rATSCs, the inhibitory concentration in half maximum (IC_50_) was around 200 µg/ml TiO_2_ NPs

MTT-based cytotoxicity test was employed to check the viability of stem cells after treatment with TiO_2_ (0–500 µg/ml) for 1, 2, and 3 days (Fig. 4). No obvious evidence of cytotoxicity was observed 1 and 2 days after treatment with different concentrations of TiO_2_ NPs, representing excellent cytocompatibility of the nanoparticles. Upon increasing the time of exposure to 3 days, cell viability was reduced. It showed that the viability of rBMSCs and rATSCs decreased in a time- and dose-dependent manner (Fig. 4).

### TiO_2_ NPs induced cell cycle distribution in rBMSCs and rATSCs

**Fig. 5:**
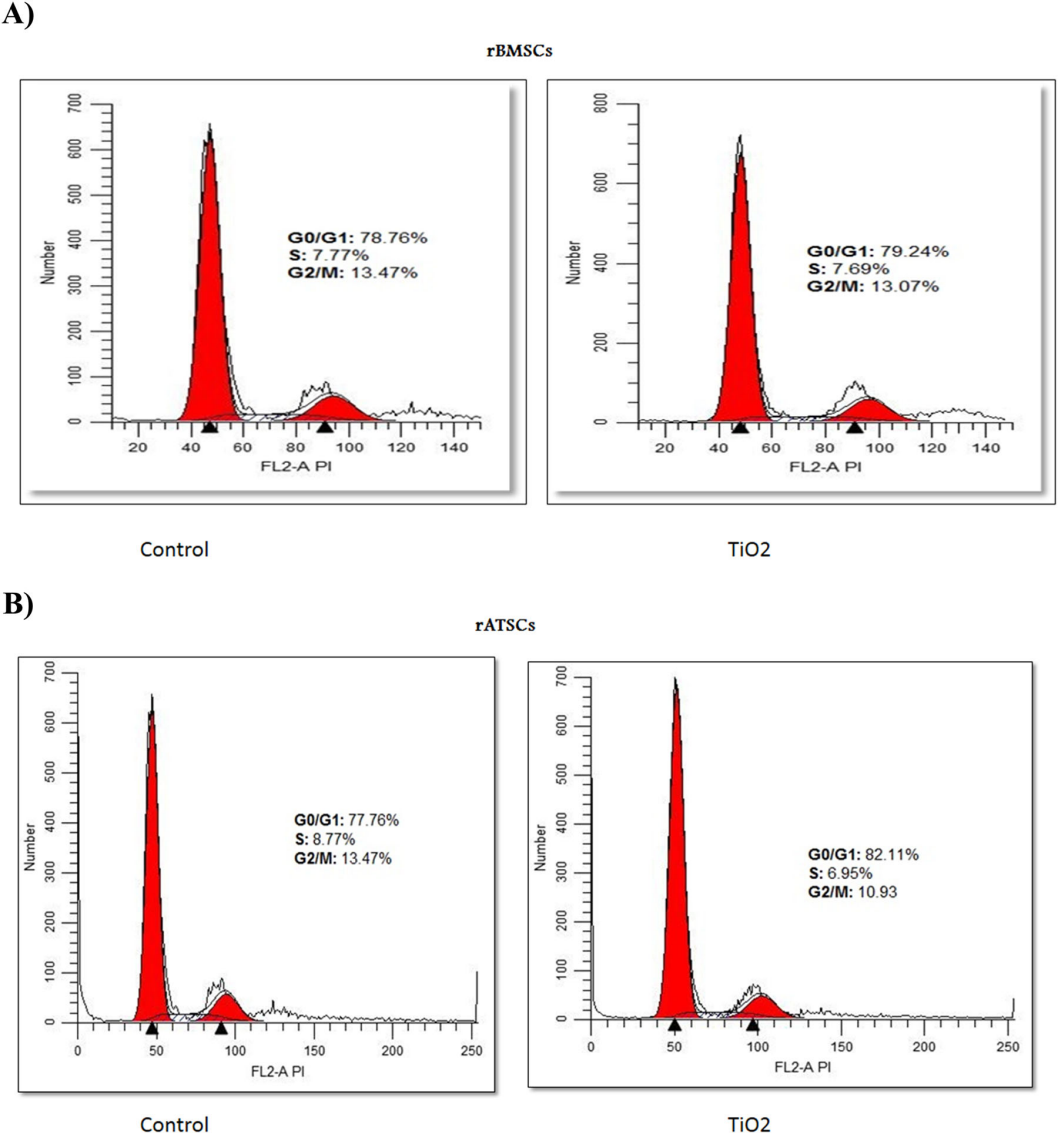
Inhibitory effect of TiO_2_ NPs on cell cycle progression of both rBMSCs and rATSCs after treating for 72 h in concentration around their IC_50_ (200 μg/ml). **A** The rBMSCs exposed to 100 µg/ml TiO_2_ presented fewer cells in S and G2/M as compared to the control group. **B** The rATSCs exposed to 200 µg/ml TiO_2_ presented fewer cells in S and G2/M as compared to the control group

Herein, cell cycle analysis was conducted to evaluate the cell cycle progression of rBMSCs and rATSCs under normal growth conditions and after being treated with NPs in concentration around their IC_50_ (100 and 200 µg/ml for rBMSCs and rATSCs, respectively). The calculation of the area under the peak was done by CellQuest software (Becton Dickinson, San Jose, CA, USA) to identify the phase within the cell cycle. The effect of TiO_2_ NPs on cell cycle distribution was studied by propidium iodide staining of the cell nucleus. The rBMSCs exposed to 100 µg/ml TiO_2_ NPs represented fewer cells in the S and G2/M phases when compared to the control group; the percentage of cells in the G0/G1 phase increased in comparison with the control group (Fig. 5). The population of cells in the S and G2/M phases reduced in the rATSCs treated with 200 µg/ml TiO_2_, while the percentage of cells entering the G0/G1 phase increased (82.11%) (Fig. 5).

### Evaluation of apoptosis using DAPI staining

**Fig. 6:**
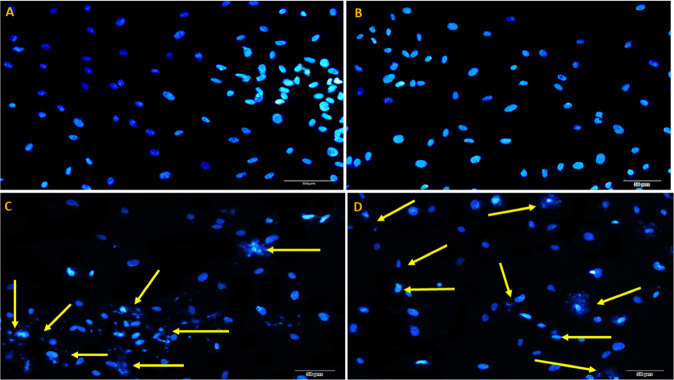
The results of DAPI staining in **A** rBMSCs and **B** rATSCs represented normal healthy chromatin. **C** TiO_2_-treated rBMSCs, **D** TiO_2_-treated rATSCs. The yellow arrows show condensed or destroyed chromatin in groups **C** and **D** cells

The morphological alteration induced by apoptosis in rBMSCs and rATSCs was studied by DAPI staining. In this regard, the density of nuclei and morphology of chromatin were observed under fluorescence microscopy after 48 h treatment by TiO_2_ NPs. The untreated rBMSCs and rATSCs (control group) showed normal nuclei (Fig. 6A, B), while the cells treated with TiO_2_ NPs represented the fragmentation of nuclei compared with untreated cells in both cells lines. In addition, the reduction in the density of the cells treated with TiO_2_ NPs is observed in comparison with the control groups indicating the engulfing of apoptotic cells through the neighboring cells or phagocytes by phagocytosis (Fig. 6C, D).

### Colony-forming results

**Fig. 7:**
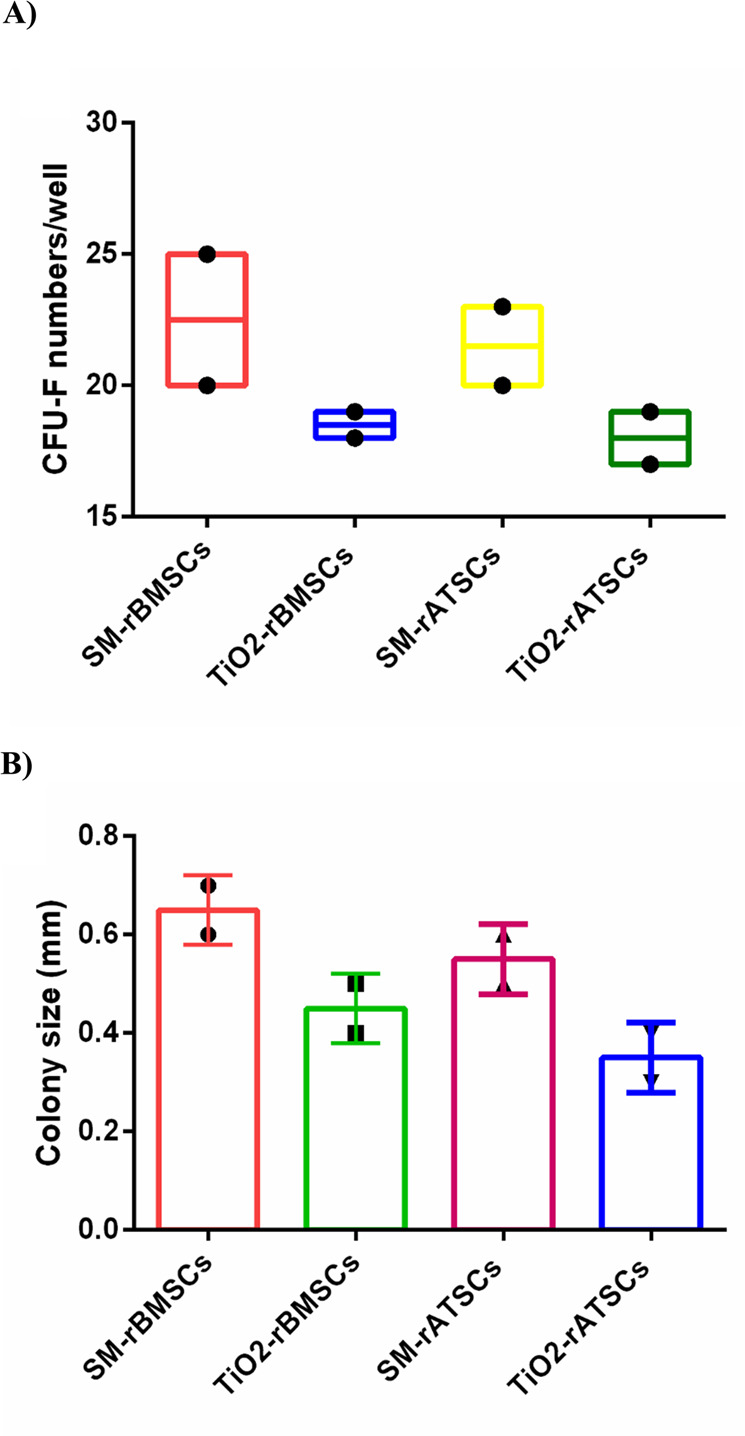
**A** The colony numbers of rBMSCs or rATSCs treated with standard medium (SM) or treated with TiO_2_. **B** The colony size of rBMSCs or rATSCs treated with SM or TiO_2_ (data was shown as mean ± SD)

Treatment with TiO_2_decreased the colony-forming unit efficiency, as evidenced by the reduced number and size of colonies from both rBMSCs and rATSCs compared to the control group (Fig. 7).

### Gene expression analysis

**Fig. 8:**
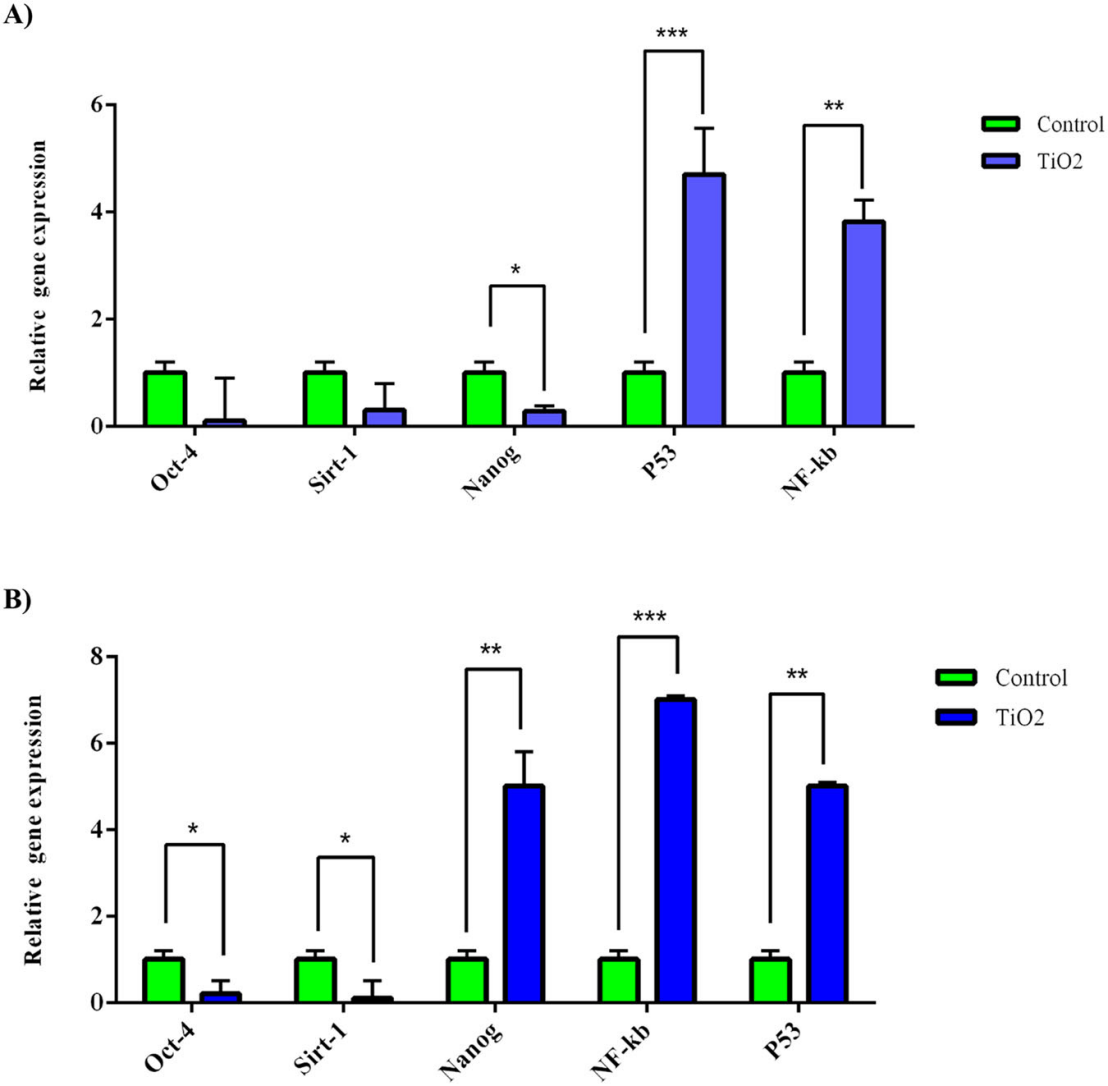
The results of aging-relevant genes expression. **A** rBMSCs exposed to TiO_2_ and **B** rATSCs exposed to TiO_2_. GAPDH was used as a housekeeping gene. *p < 0.05, **p < 0.01, ***p < 0.001

**Fig. 9:**
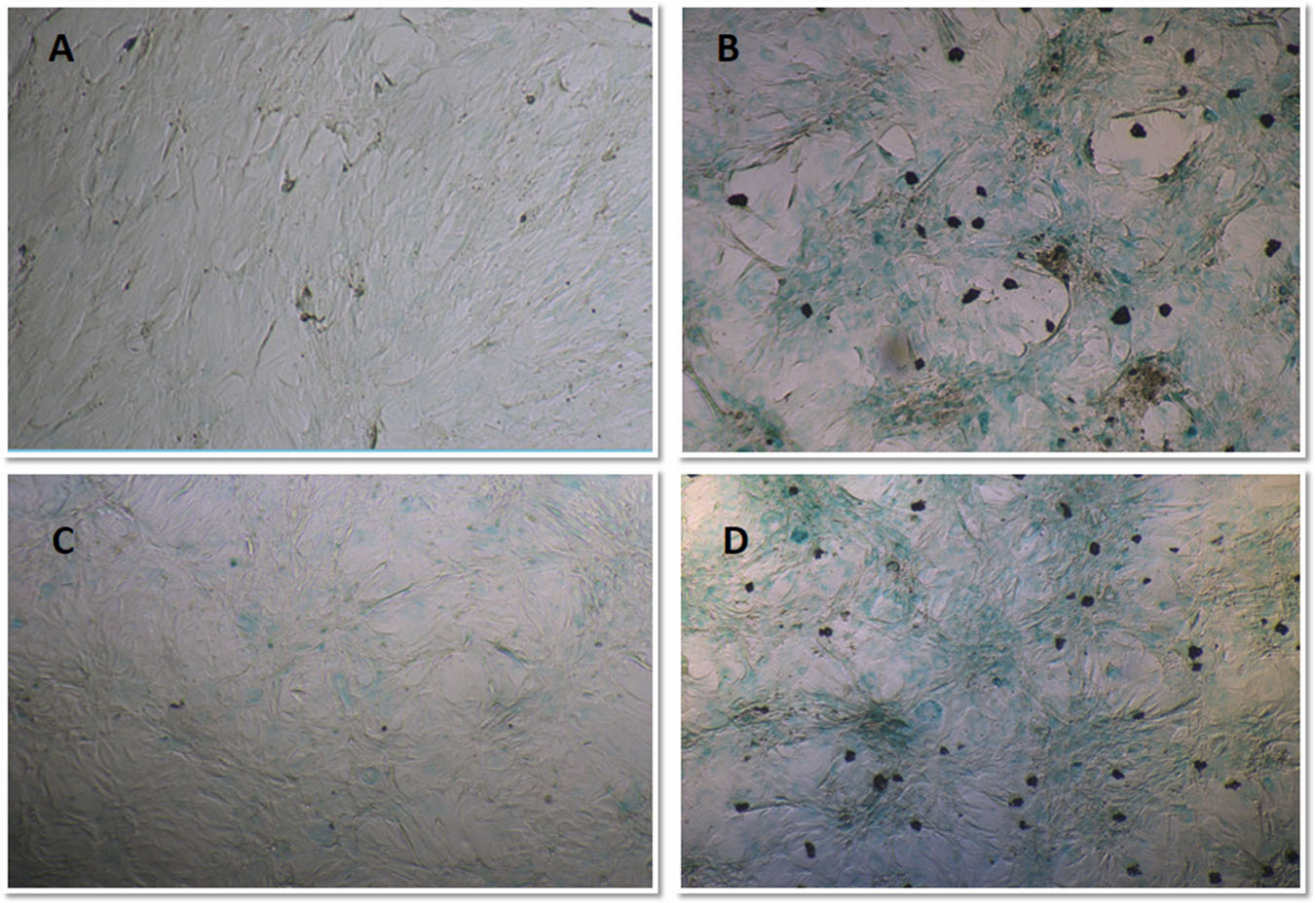
Senescence-associated-β-galactosidase (SA-β-gal) staining in the rATSCs and rBMSCs at 100 and 200 µg/ml TiO_2_ NPs. SA-β-gal-positive cells could be observed more frequently in the **B** rATSCs and **D** rBMSCs treated with 100 and 200 µg/ml TiO_2_NPs, respectively compared to control groups (**A** and **C**)

The relative expression of P53 and NF-kB as aging-relevant genes was evaluated 48 h after treatment with 100 and 200 µg/ml TiO_2_NPs for rBMSCs and rATSCs, respectively. A significant up-regulation of the NF-kB gene was observed at the concentration of 200 µg/ml (*p* ≤ 0.001) compared to other groups (Fig. 8A, B). However, there was a significant (*p* ≤ 0.05) up-regulation in both rBMSCs and rATSCs at 100 µg/ml in comparison with the control group (Fig. 9A, B). As shown in Fig. 8, a significantly higher expression of P53 (*p* ≤ 0.0001) ‏was found in both rBMSCs and rATSCs after treatment with 100 µg/ml TiO_2_ NPs compared to other groups. When compared to the control group, a significant up-regulation (*p* ≤ 0.001) was also detected at 200 µg/ml concentration (Fig. 8).

Relative expression of Sirt1 ‏ and Nanog ‏as anti-aging genes‏ ‏was also measured. The results showed significant down-regulation of the Nanog gene in both rBMSCs and rATSCs compared to control groups at both 100 and 200 µg/ml concentrations (*p* ≤ 0.0001) (Fig. 8). Significant down-regulation (*p* ≤ 0.001) was also observed in the expression of the Sirt1 gene in comparison with the controls (Fig. 8).

### β-galactosidase in situ assay

Beta-galactosidase enzyme activity was measured to assess the effect of TiO_2_ NPs on the senescence of MSCs. The results, depicted in Fig. 9 represented a higher β-galactosidase activity (the TiO_2_-treated cells with positive green staining of cell areas compared to the controls).

## Discussion

One of the main issues in nanotechnology and nanoscience is the more toxicity of nanoparticles in comparison with the large-sized particles, as a result of their extensive surface area and enhanced cellular uptake [27]. Due to the wide application of TiO_2_ NPs, the exposure of human beings to these nanoparticles is going to be increased. As a result, the TiO_2_ NPs have been widely studied regarding their potential toxicity to human health.

The present study evaluated the potential toxicity of different concentrations of TiO_2_ nanoparticles on rat-derived stem cells. The results indicated that TiO_2_ NPs cause toxic effects on the growth, clonogenicity, stemness, cell cycle, DNA fragmentation, and apoptosis of rBMSCs and rATSCs.

In this study, we used bone marrow and adipose tissue derived from rats as model cells due to the potential application of the MSCs, especially from bone marrow, in the toxicity study that was evidenced in another study [18]. After isolation of model cells, both rATSCs and rBMSCs were studied to confirm the characteristics of MSCs, surface CD markers, osteogenic differentiation, and morphological phenotype.

TiO_2_ NPs were also characterized in terms of size and morphology utilizing DLS and SEM, respectively. As shown in the results section, the particles are irregular rock-like structures with large variations in size ranging from a few nanometers to around 2.6 microns. The colorimetric assay indicated a decline in proliferation, colony-forming, and cytotoxic properties. Generally, the cytotoxicity of nanostructured materials is dependent on the NPs concentration, type and structure, time of exposure, method of interaction with cells, and so on [28]. Our results are in line with those observed by Ghanbary et al. on the cytotoxicity of TiO_2_ nanoparticles in human monocytes [29]. The cytotoxic effect of TiO_2_ NPs on monocytes is proved to be related to intracellular ROS generation, collisions of mitochondrial membrane potential, lipid peroxidation, glutathione depletion, and lysosomal membrane damage [29, 30].

The in situ assay of β-galactosidase revealed cellular SA-β-Gal enzyme activity in the cells treated with 100 and 200 µg/ml of TiO_2_ NPs in both rBMSCs and rATSCs. In addition, extreme up-regulation of P53 and NF-kB genes was observed in those cells. P53 is regulated directly or indirectly via PKB/AKT kinase as the main factor of insulin/insulin-like growth factor (IGF)-1 signaling (IIS). Signaling of PKB/AKT is also pro-aging by NF-kB transcription factor [31, 32]. The NF-kB motioning pathway is suggested as another important aging mediator. This pathway is triggered by oxidative stress, genotoxicity, regulation of cytokine expression, inflammatory stress, regulating apoptosis genes, growth factors, cell senescence, cell cycle progression, and inflammation. NF-kB transcriptional activity is enhanced with aging in many of the tissues. NF-kB is related to various age-linked degenerative diseases such as osteoporosis, Alzheimer’s disease, and diabetes. NF-kB is a general responder to a wide range of stress stimulation and plays a main role in aging. NF-kB is directly involved in the aging process [33, 34]. Therefore, the increased activity of both p53 and subsequently NF-kB is related to the aging-inducing effect of TiO_2_. It is in agreement with the results of the present study on P53 and NF-kB as promoting aging factors [32, 35]. Moreover, down-regulation of anti-aging-related genes, as well as Nanog and Sirt-1, was detected in the stem cells treated with (200 µg/ml) of TiO_2_ NPs. Nanog prevents the aging effects on the proliferation of MSC and is noticeably up-regulated in DNA damage repair, DNA replication, and cell cycle [36]. In the previous studies, Nanog has been shown to enhance somatic and stem cell proliferation and self-renewal by regulating cell cycle, stemness, and senescence pathways [37]. Nanog is a homeodomain of transcription factor which not only has the potential to postpone aging but also can reverse aging [36, 38, 39]. There has been scientific evidence that Sirt1 activation disorders are associated with the onset of aging. Sirt1 has been shown to have a role in nutrient homeostasis and energy maintenance, thereby exerting its anti-aging effect due to its role in body metabolism [40, 41]. The main role of sirtuin proteins in promoting stress resistance and survival, which leads to longevity [42]. Recently, the anti-aging effects of caloric restriction have been simplified, and Sirt1 has been recognized as a caloric restriction-associated anti-aging molecule [42].

TiO_2_ NPs enter the body via many routes, causing toxicity via deposition in different organs and induction of cell apoptosis They also change the expression of hormones and damage the body organs [43]. In addition, TiO_2_ NPs becomes a substance to generate reactive oxygen species (ROS) intermediates in the biological systems because it is highly photoreactive [44]. ROS intermediates have harmful effects on cell viability and functions and subsequently stimulate innate immune responses, which results in inflammation and genotoxicity [45].

There are different studies on the suppression effect of TiO_2_ NPs on the cell cycle progression of various cell types [46]. Hou et al. investigated the effect of the highest concentrations of TiO_2_ on different phases of the mouse MSCs cycle (S, G1, G2). They represented a reduction in the number of cells in the S and G2 phases [47]. According to our cell cycle assay, TiO_2_ NPs induce apoptosis and inhibit the cell cycle, entering the syncretization phase (S) of a cell cycle. In cosmetic products, TiO_2_ nanoparticles are found as oil-in-water emulsions, which can easily penetrate the skin [48] and exhibit their negative effects.

DAPI staining of rATSCs and rBMSCs represented the degradation, fragmentation, and decrease in the density of the nuclei of the cells treated with TiO_2_ NPs. Based on our results, TiO_2_ NPs can also enter the nucleus and damage DNA directly or indirectly via alterations in gene expression [49]. Wamer et al. depicted that, the treatment of human skin fibroblasts with TiO_2_ NPs causes damages to the RNA and DNAs. According to their thesis, damaged RNA indirectly affects gene expression [50]. The pathological examination of the effects of TiO_2_ NPs in the liver and kidney has revealed that they are deposited and cause necrosis, cell apoptosis, and fibrosis in the liver, and glomerular swelling in the kidney [9, 48, 50, 51].

## Conclusion

In this study, the effect of TiO_2_ NPs on both rat MSCs from bone marrow and adipose tissues was explored. The nanoparticles were studied in terms of morphology and size. The results represented that they are irregular rock-like structures with large variations in size ranging from a few nanometers to around 2.6 microns. Until now, a few studies have been conducted on the destructive effects of TiO_2_ NPs in living organisms, and studies on the toxicity of TiO_2_ NPs are still in the beginning phases. Because of the widespread use of TiO_2_ NPs in all areas of human life, it is essential to study their profound and fundamental toxic effects on each organ and body cell. Based on the results obtained from the MTT assay, TiO_2_ NPs showed cytotoxicity in both rBMSCs and rATSCs in a time- and dose-dependent manner. Also, the DAPI staining study and cell cycle analysis represented the induction of apoptosis in both cell types. Furthermore, constant exposure of tissues and organs to TiO_2_ NPs may lead to the accumulation of these particles. This accumulation results in the reduced capacity of MSCs for growth, colony formation, and triggers cellular damage and senescence. Such findings should be considered when using massive doses and long time consuming of TiO_2_ NPs. Considering the aforementioned results and taking into account the frequent exposure of human beings with TiO_2_ NPs, we can conclude their adverse effect on human health.
